# Immunotherapeutic allogeneic dendritic cell and autologous tumor cell fusion vaccine alone or combined with radiotherapy in canine oral malignant melanoma is safe and potentially effective

**DOI:** 10.3389/fvets.2024.1397518

**Published:** 2024-08-20

**Authors:** Yuan-Yuan Xia, Albert TaiChing Liao, Ru-Min Liu, Shu-Ya Yang, Chien-Chun Kuo, Chiao-Hsu Ke, Chen-Si Lin, Jih-Jong Lee

**Affiliations:** ^1^Department of Veterinary Medicine, School of Veterinary Medicine, National Taiwan University, Taipei, Taiwan; ^2^Graduate Institute of Veterinary Clinical Science, School of Veterinary Medicine, National Taiwan University, Taipei, Taiwan; ^3^National Taiwan University Veterinary Hospital, National Taiwan University, Taipei, Taiwan

**Keywords:** canine oral melanoma, immunotherapy, dendritic cell fusion vaccine, radiotherapy, immunotherapy combined with radiation

## Abstract

**Introduction:**

Immunotherapy represents a promising breakthrough in cancer management and is being explored in canine melanomas. Dendritic cells (DCs) play a crucial role in priming T-cell-mediated immune reactions through the antigen-presenting function. Combining immunotherapy and radiation therapy may generate more substantial anti-cancer efficacy through immunomodulation.

**Objectives:**

Our research reported a preliminary result of the safety and outcome of a kind of immunotherapy, the allogeneic dendritic cell and autologous tumor cell fusion vaccine, alone or in combination with hypofractionated radiation therapy, in canine oral malignant melanoma.

**Methods:**

Two groups of dogs with histopathological diagnoses of oral malignant melanoma were recruited. In group 1 (DCRT), dogs received a combination of DC fusion vaccine and radiotherapy. In group 2 (DC), dogs received DC fusion vaccine alone. DC vaccination was given once every 2 weeks for four doses. Radiotherapy was performed weekly for five fractions. Dogs that received carboplatin were retrospectively collected as a control group (group 3).

**Results:**

Five dogs were included in group 1 (two stage II, three stage III), 11 in group 2 (three stage I/II, eight stage III/IV), and eight (two stage I/II, six stage III/IV) in the control group. Both DC and DCRT were well-tolerated, with only mild adverse events reported, including mucositis, gastrointestinal discomfort, and injection site reactions. The median progression-free intervals in groups 1, 2, and 3 were 214 (95% CI, NA, due to insufficient data), 100 (95% CI, 27–237), and 42 days (95% CI, NA-170), respectively, which were not significantly different. The 1-year survival rates were 20, 54.5, and 12.5% in groups 1, 2, and 3. Dogs in the DCRT group exhibited significantly higher TGF-β signals than the DC group throughout the treatment course, indicating a possible higher degree of immunosuppression.

**Conclusion:**

The manuscript demonstrated the safety of dendritic cell/tumor cell fusion vaccine immunotherapy, alone or in combination with radiotherapy. The results support further expansion of this immunotherapy, modification of combination treatment and protocols, and investigation of combining DC vaccine with other treatment modalities.

**Clinical trial registration:**

Preclinical Trials, PCTE0000475.

## 1 Introduction

Canine oral malignant melanoma (OMM) is the most common tumor in the canine oral cavity and its management remains challenging due to its propensity for local invasion and distant metastasis (1). Surgery and radiation therapy (RT) provide the most effective tumor controls which primarily target local tumor invasion. Hypofractionated radiation, as reported in several studies, has been commonly used in canine OMM, demonstrating an overall 81–100% tumor response rate with tolerable side effects. Reported median survival times of dogs with OMM undergoing RT alone range from 171 to 307 days, while additional systemic treatments appeared to have minimal impact on survival (2–5). Considering the high rate of distant metastasis, systemic treatment against canine OMM is imperative. Conventional chemotherapy utilizing platinum-based agents, however, only offered a modest 28–37% response rate (6, 7). In our department, using chemotherapy in dogs with OMM lacking wide-margin surgery resulted in a 12.5% response rate and a median overall survival of 6 months (8). Evidence of therapeutic effects in canine OMM is also limited for tyrosine kinase inhibitors (TKIs) (9, 10). On the other hand, immunotherapy, aiming to activate and modulate the immune system, is the fourth pillar in both human and veterinary cancer management.

At the time of this manuscript preparation, two commercialized immunotherapeutic drugs were approved for treating canine malignant melanoma. The first is the US FDA and USDA-approved DNA vaccine Oncept^®^, which expresses xenogeneic human tyrosinase and is applied for dogs with stage II/III oral melanoma after surgical control since 2007. Theoretically, the DNA product was designed to induce an immunostimulatory function, but the clinical experiences were controversial (11, 12). Although some long-term survivors were observed, and it remained an option due to the vaccine’s general safety, more solid evidence of efficacy was required. The second, the immune checkpoint inhibitor (ICI), represents a promising immunotherapeutic breakthrough in human and canine cancer fields. In late 2023, the commercialized canine anti-programmed cell death receptor-1 (PD-1) antibody Gilvetmab was conditionally approved by the USDA for canine mast cell tumor and malignant melanoma treatment, and further clinical studies are underway to investigate its toxicity and efficacy. Other research on canine ICIs showed some survival benefits in end-stage OMM dogs. In the study conducted by Igase et al. (13) the anti-PD-1 treatment induced a 26.5% response rate. And Maekawa et al. (14) reported that the anti-PD-ligand-1 (PD-L1) treatment resulted in a significantly prolonged survival of 143 days compared to a historical control.

Our team has constructed a cancer immunotherapeutic vaccine by fusing autologous cancer cells with allogeneic dendritic cells (DCs) and re-injecting the fusion product subcutaneously into tumor-bearing dogs. This approach aimed to capitalize on the great antigen-presenting and processing ability of DCs, to prime a specific anti-tumor immune response involving both CD4 and CD8 T cells (15). Previously, Gyorffy et al. (16) reported a successful generation of autologous bone-marrow-derived DCs (BMDCs) from three melanoma dogs and a healthy dog. After infection with the human xenoantigen gp100, the DC-product was re-injected into dogs, with a combination of RT. Although the case number was low, two out of three melanoma dogs lived for over 20 months. However, because the DC function might be defective during tumor proliferation process (15), an allogeneic DC source from healthy young adult dogs was preferred by our team. Based on this conception, the allogeneic BMDC/autologous tumor cells fusion vaccine was conducted by our team and re-injected into transmissible venereal tumor (TVT)-inoculated beagles. The vaccination significantly slowed tumor growth rate and induced earlier self-regression without significant side effects. Increased MHC expression on tumor cells, enhanced TVT-specific cytotoxicity and natural killer cell activity, as well as increased interferon (IFN)-γ production, were observed in the vaccinated group (17). The DC generation method in this experiment was successfully repeated in serial studies, as confirmed by morphology and cell phenotypes (18, 19). Another team used allogeneic or autologous DCs and fused them with canine mammary gland tumor cell lines to develop fusion vaccine products. The two kinds of vaccines were injected into laboratory beagles, resulting in cytotoxic T-lymphocyte reaction and specific anti-tumor IgG detection, respectively (20, 21). These previous basic and clinical data support us in further investigating the clinical efficacy of DC-based vaccines in cancer-bearing dogs.

While we treat cancer using the above strategies separately, whether those therapies could be combined to enhance treatment efficacy was asked. Combinatorial therapies have already been applied in chemotherapy and RT (22), as some chemotherapy agents are radiosensitizers. Combining immunotherapy and RT is gaining attention in recent cancer research. Radiation can induce both local and systemic anti-tumor immune reactions, and there were occasional reports of the “abscopal effect” in humans, describing the phenomenon of regression of unirradiated lesions (23). However, RT also causes the accumulation of several immunosuppressive cells and cytokines, resulting in a negative impact on the immune system (23, 24). Many pre-clinical and clinical studies are working on combining RT and immunotherapy using ICIs, with some encouraging pre-clinical evidence and some controversial clinical experiences (23–29). To further explore the clinical efficacy, more issues about the exact treatment sequence, RT fractionation and treatment resistance, should be addressed. In veterinary research, only a few published studies focused on this topic. Canter et al. (30) applied RT and subsequent intra-tumoral natural-killer (NK) cell transfer in a canine osteosarcoma mouse model and clinical patients. Delayed tumor growth and enhanced NK cell homing to the tumor were observed in the mouse model. Fifty percent of clinical osteosarcoma dogs were metastasis-free after 6 months, with acceptable side effects (30). Deguchi et al. (31) retrospectively analyzed dogs with stage IV OMM being treated with anti-PD-L1 and hypofractionated RT, and a 55.6% clinical benefit rate was reported in dogs receiving RT ≤ 8 weeks before anti-PD-L1 treatment, which was significantly higher than the group of dogs having ICIs alone. Boss et al. (32) recruited dogs with spontaneous tumors and treated them with stereotactic body radiotherapy (SBRT) alone or combinatorial OX40/TLR immunotherapy. The latter group of dogs had decreased tumor-infiltrating regulatory T cells (Tregs) and tumor macrophages, as well as a significantly increased serum interleukin (IL)-7 concentration (32). Besides, Magee et al. (33) employed a kind of immune-radiotherapy composed of external beam radiotherapy, intra-tumoral cytokine and targeted radionuclide. The treatment was well-tolerated and could induce tumor microenvironment modulations (33). As dogs are great animal models in cancer research, more studies are warranted exploring the immunotherapy and RT combinational treatment modality.

Based on the information above, our manuscript outlines a pilot clinical study investigating the use of the DC/tumor fusion vaccine alone or in combination with RT in dogs with OMM. The study aimed to address two questions: (1) the safety of the DC fusion vaccine alone and in combination with RT, and (2) the outcome of the DC fusion vaccine and the combinatorial treatment.

## 2 Materials and methods

### 2.1 Study design and patient recruitment

The study was executed at National Taiwan University Veterinary Hospital Animal Cancer Treatment Center, and was a single-center, open-label pilot study. Client-owned dogs were enrolled into Group 1 (DCRT) or Group 2 (DC), which was decided by the owners based on the clinicians’ suggestions on possible treatment options.

Group 1 (DCRT): due to the COVID-19 pandemic, radiation therapy for small animals has been unavailable in our area since 2020. Therefore, only dogs from 2019 to 2020 that received concurrent radiation therapy and dendritic cell immunotherapy were recruited. Dogs in this group should have histopathological diagnoses of oral malignant melanoma. Dogs were not required to be treatment-naïve but should fail previous treatment. For surgical procedures, either tumor debulking or biopsy, or wide-margin surgery (e.g., partial or total maxillectomy or mandibulectomy) was acceptable. Whether to perform a regional lymphadenectomy was determined by the clinician. Dogs should be clinically staged based on the WHO TNM staging system (Table 1). Dogs in stage I-III were included because surgery and RT would not be strongly recommended by clinicians for stage IV dogs considering the cost and risks of repeated anasthesia. During the staging process, tumor size was measured by caliper or by head computed tomography (CT) scan. The ipsilateral mandibular lymph nodes or any enlarged mandibular or retropharyngeal lymph nodes were defined as regional lymph nodes (RLNs), and metastasis was surveyed through histopathology or cytology, or was suspected by radiologists through CT. Pulmonary metastasis was screened by thoracic CT and soft tissue attenuation lesions would be presumed to be metastasis without further cytology or pathology confirmation. Basic blood tests, including a complete blood count (CBC) and biochemistry, were obtained before enrollment. Dogs with severe liver or renal insufficiency or autoimmune disease were not eligible to be included. Other examinations, such as urinalysis and abdominal ultrasound, were not required in each patient but were determined by the attending clinician. Concurrent use of steroids was not allowed. The study was fully reviewed and approved by the National Taiwan University Institutional Animal Care and Use Committee (approval no. NTU-109-EL-00106). All the owners were informed of the study details before enrollment, and informed consent was obtained.

**Table 1:** WHO-based TNM clinical staging system of canine oral malignant melanoma.

T: Primary tumor		N: Regional lymph nodes		M: Distant metastasis	
T1	Tumor ≤2 cm in diameter	N0	No evidence of regional node involvement	M0	No evidence of distant metastasis
T2	Tumor 2–4 cm in diameter	N1	Histologic/cytologic evidence of regional node involvement	M1	Evidence of distant metastasis
T3	Tumor >4 cm in diameter	N2	Fixed nodes		
Stage I = T1 N0 M0					
Stage II = T2 N0 M0					
Stage III = T3 N0 M0 or T2 N1 M0					
Stage IV = Any T, any N, and M1					

Group 2 (DC): dogs that received dendritic cell immunotherapy without RT were recruited from 2019 to 2022. The diagnosis criteria, surgical procedures, examination of primary oral mass and definition of RLN, decisions on regional lymphadenectomy, staging criteria, and exclusion criteria were the same as in Group 1. During the initial staging process, if the initial chest X-ray survey revealed evidence of pulmonary metastasis, the dog would be excluded because surgical procedures would not be strongly suggested. If the pulmonary metastatic lesion was tiny and could only be detected by a CT, the dog was still allowed to be enrolled. Therefore, dogs in stage I-III and early stage IV were included. Similarly, baseline CBC and biochemistry were obtained before enrollment, and other clinical examinations were not required but were determined by the clinician. Concurrent use of steroids was not allowed. The study was fully reviewed and approved by the National Taiwan University Institutional Animal Care and Use Committee (approval no. NTU-110-EL-00134). All the owners were informed of the study details before enrollment, and informed consent was obtained.

Control group: because neither radiotherapy nor commercialized Oncept immunotherapy is available in our area currently, a group of dogs (Group 3) with histopathologically diagnosed OMMs who received carboplatin in our department was used as a control group (data of this population was reported as a part of our previous work) (8), to compare the treatment efficacy preliminarily. Patients’ information, treatment, and outcome details were recorded. Evaluation criteria for clinical stage, RLN, and distant metastasis were the same as those in groups 1 and 2. None of the dogs received wide-margin surgery or RT. All of the patients had maximum-tolerated-dose carboplatin, treatment dose and interval were determined by the attending clinician.

### 2.2 Dendritic cell immunotherapy manufacturing

The dendritic cell immunotherapy utilized autologous tumor cells and allogeneic dendritic cells, which were fused *in vitro* into a vaccine product. A detailed process was described previously (17, 34).

Tumor cell preparation: freshly biopsied or removed tumor tissue was suspended and was mechanically crushed and separated into single cells in a sterile stainless steel mesh, with phosphate-buffered saline (Simply, GeneDireX, Taipei, Taiwan) solution with 5% antibiotics of Penicillin–Streptomycin-Amphotericin B (P/S/A, Simply, GeneDireX, Taipei, Taiwan). At least a 1*1*1 cm tumor sample was requested to obtain sufficient tumor cells, but it was encouraged to be as large as possible. The total cell count should be at least 4 × 10^7^, and the cells were preserved in cryogenic tubes. Each tube contains around 1 × 10^7^ cells. All the cells were checked microscopically to ensure no bacterial or fungal infection and were then stored in nitrogen liquid until vaccine preparation.

Dendritic cell generation: peripheral blood was collected from healthy dog donors. Mononuclear cells (PBMCs) were isolated by gradient centrifugation using Ficoll–Hypaque (density 1.077, Cytiva, Uppsala, Sweden) at 400 g for 35 min at room temperature. The buffy coat was extracted and centrifugated at 500 g for 15 min at 16°C and was washed twice using PBS. The obtained PBMCs were cultured in RPMI 1640 medium (Simply, GeneDireX, Taipei, Taiwan) with 10% donor dog serum and 1% P/S/A for 24 h (day 1) for cell adherence. From the second day to the seventh day, the culture medium was changed to RPMI 1640 with 10% fetal bovine serum (FBS, Gibco, Thermo Fisher Scientific, Massachusetts, United States), 1% P/S/A, and IL-4, GM-CSF and Flt-3 L (all from R&D System, Minnesota, United States) to induce immature dendritic cell differentiation. On day 8, additional lipopolysaccharide (LPS, Merck KGaA, Darmstadt, Germany) was added to stimulate dendritic cell maturation. On day 11, the mature dendritic cells were harvested and every 1 × 10^7^ cell was preserved in a cryogenic tube and stored in nitrogen liquid until vaccine preparation.

Fusion vaccine preparation: 1 × 10^7^ tumor cells and 1 × 10^7^ dendritic cells were thawed and recovered 1 day before cell fusion. The tumor cells and dendritic cells were mixed and fused by adding 1 mL of polyethyleneglycol (PEG, Jena Bioscience GmbH, Jena, Germany) to the resuspended cell pellet during 2-min stirring. Based on previous studies, the fusion rate could reach 60%. The fusion product was cultured in RPMI 1640 with 10% FBS, 1% P/S/A, IL-4, and GM-CSF for 3 days before treatment. On the treatment day, the fusion product was treated with 15 ug/mL mitomycin (BOC Science, New York, United States) and was then resuspended in 400 μL 0.9% normal saline. The fresh fusion vaccine product should only be valid for use on the same day.

### 2.3 Radiation therapy

A hypofractionated radiation therapy protocol was used and dogs were treated weekly. Radiation was delivered by a 6 MV linear accelerator (Synergy, 500 MU/min, Elekta, Stockholm, Sweden). Pre-treatment cone-beam CT (Discovery CT 590, GE, 16 slices) was performed 1 week before treatment for treatment planning. Patient positioning for the CT scan was determined by the attending clinician, and a thermoplastic facial mask was used for immobilization. Gross tumor volume (GTV) was defined as primary tumor volume. Whether to include RLNs in the treatment field was determined by the attending clinician. For the primary tumor, radiation therapy was prescribed with 8–8.5 Gy per fraction, for a total of 40–42.5 Gy. While for the RLN, the dosage was 7–8 Gy per fraction. General anesthesia was performed by clinical veterinarians from the National Taiwan University Veterinary Hospital. A follow-up CT scan was arranged 1 month after finishing treatment.

### 2.4 Treatment protocol and schedule

The freshly harvested dendritic cell/tumor cell fusion vaccine was given subcutaneously at the lateral cervical region ipsilateral to the tumor, between mandibular and prescapular lymph nodes.

**Figure 1:**
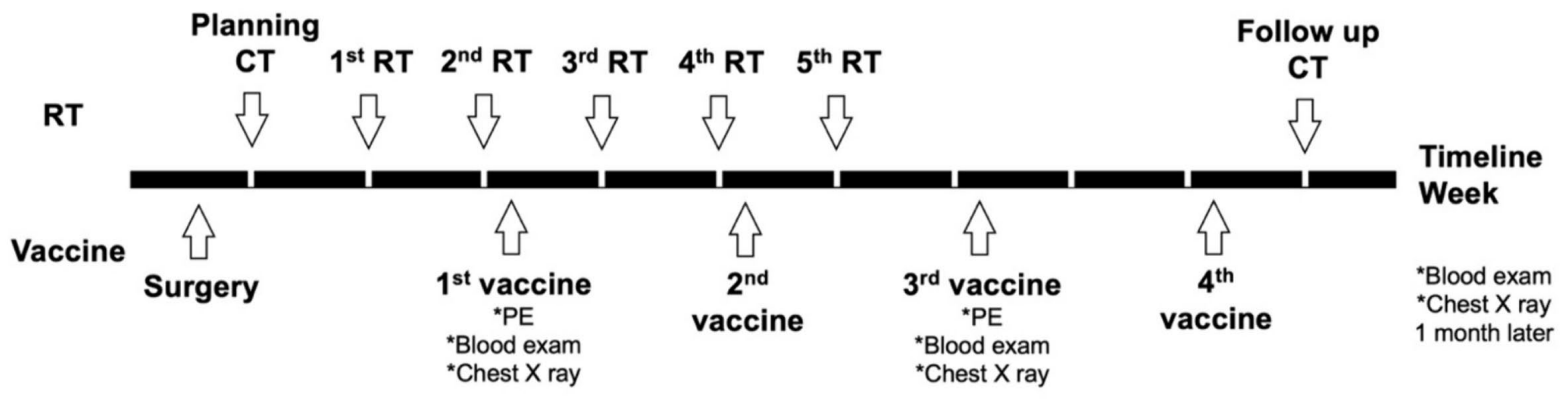
Planning treatment schedule of the dogs in the DCRT group. Briefly, surgery was performed for DC vaccine sampling and debulking, and CT planning for RT was performed in the same week of surgery. The first RT started 1 week after CT planning; the first DC vaccination started 2 days after the second RT. RT was scheduled weekly for a total of five treatments; the DC vaccine was given every other week for a total of four doses. PE, physical examination.

For the group 1/DCRT group, the treatment schedule is summarized in Figure 1. Briefly, the primary tumor sample was biopsied for vaccine preparation, and the patient would receive CT planning in the same week. RT would be started next week and proceeded weekly for five treatments. The DC vaccine would be given 2–3 days after the second RT treatment due to the time needed for manufacturing. The DC vaccine would be prescribed every 2 weeks for a total of four doses. A follow-up CT scan was arranged 4 weeks after the fifth RT, and would be in the same week as the last DC vaccination.

For the group 2/DC group, patients had their tumors removed or biopsied for vaccine preparation. Patients would receive the DC vaccine 1–2 weeks after surgery once the surgical wound healed well. The vaccination would be prescribed once every 2 weeks, and a total of four doses were planned.

### 2.5 Response and adverse event evaluation

Tumor response was evaluated based on the Veterinary Cooperative Oncology Group Response Evaluation Criteria in Solid Tumors v1.0 (35) and was defined as complete remission (CR), partial remission (PR), stable disease (SD) and progressive disease (PD). If the best response was SD, the duration should be at least 4 weeks. A clinical benefit rate was calculated as the percentage of patients who achieved CR/PR/SD. The response rate was defined as the percentage of dogs achieving CR and PR.

Radiation toxicity was evaluated based on the toxicity criteria of the veterinary radiation therapy oncology group (36) and was assessed and graded at each treatment and recheck. Radiation side effects were defined as acute (within 6 months) or delayed (>6 months). If acute mucositis occurred and affected the patient’s quality of life, nonsteroidal anti-inflammatory drugs were prescribed to alleviate clinical signs.

Immunotherapy toxicity was evaluated based on clinical signs, physical examination, blood, and imaging examinations. Physical examination was performed at each treatment and recheck. Essential CBC and biochemistry, and 3-view chest radiographs were checked before the first and third vaccinations, and 1 month after the fourth vaccination. To classify the adverse events more specifically, the Veterinary Cooperative Oncology Group—Common Terminology Criteria for Adverse Events (VCOG-CTCAE) v1.1 and v2 (37, 38) were used as reference criteria, which were also used for chemotherapy-caused adverse event evaluation.

Patient follow-up and re-staging examinations were arranged monthly for 3 months and every 3 months after.

### 2.6 Peripheral blood plasma cytokine concentration analysis

In group 1 and group 2, blood samples from individual patients were collected in EDTA tubes before the first and third vaccination, and 4 weeks after the fourth vaccination. Plasma was preserved at −20°C until analysis. The plasma cytokine analysis was proceeded by using the commercial ProcartaPlex Dog Cytokine/Chemokine Panel 11 plex (including IFN-γ, IL-10, IL-12, IL-2, IL-6, IL-8, MCP-1, SCF, TNF-α, VEGF-A, NGF-β) and ProcartaPlex Dog TGF-beta-1 Simplex kit and analyzer (Invitrogen, Thermo Fisher Scientific, Massachusetts, United States).

### 2.7 Statistical analysis

Patient’s signalment, tumor information (oral tumor size, location, bony invasion), RLN status, clinical stage, mitotic count under histopathological evaluation, surgical procedure, residue tumor status before DCRT or DC treatment, tumor response during treatment (i.e., PD or not), and other anti-cancer treatments before enrollment or after disease progression (i.e., chemotherapy, targeted therapy, other immunotherapies), were recorded. Progression-free interval (PFI) and overall survival (OST) were recorded. PFI was calculated from the day the studied treatment started to the day of disease progression or other treatment initiation. OST was defined as disease-specific survival and was calculated from the start of treatment to the time of tumor-related death. For patients who received additional treatments after PD or due to their owners’ insistence, the OST would be recorded from the day the studied treatment started to the time the other treatments were initiated, and then the data would be censored from survival analysis. If disease progression was not confirmed, or the death was unrelated to melanoma, the data would still be recorded but would be censored from PFI or OST analysis. Local recurrence was defined as a cytologically or histologically diagnosed melanoma that recurred at the original site or RLNs after treatment. Distant metastasis was detected by radiography or ultrasonography. Categorical variables were compared using Fisher’s exact test. Continuous variables were analyzed by the Mann–Whitney test. Cytokines at different time points within a single group were analyzed by the Wilcoxon test. Cytokines at different time points between the DCRT and DC groups were analyzed by multiple Mann–Whitney tests with an adjusted *p*-value by using the Holm-Sidak method (setting *α* = 0.05) to control the type I error rate when performing the multiple comparisons. PFI and OST were described by Kaplan–Meier curves with 95% confidence interval obtained directly from the graphs, and were compared by Log-rank test. All statistical analyses were performed by GraphPad Prism (RRID: SCR_002798), version 10.0, GraphPad Software, San Diego, California United States.^1^ A *p* < 0.05 was considered statistically significant.

## 3 Results

### 3.1 Patients’ characteristics

Group 1/DCRT group: five dogs were prospectively enrolled. Detailed patient characteristics are summarized in Table 2. The median age of the five dogs was 12 years old (interquartile range/IQR, 11–13). The median body weight was 5.8 kg (IQR, 4.3–13). The median oral tumor size was 3.0 cm (IQR, 2.5–3.4). Two dogs (40%) had mandibular tumors while the other three (60%) had maxillary tumors. Two dogs were stage II (40%), and three were stage III (60%). Four out of five (80%) dogs had metastatic RLNs. Before enrollment, four dogs were treatment-naïve; one had received metronomic chemotherapy for 2 weeks with macroscopic disease, without obvious response. None of the dogs received wide-margin surgery. All the dogs had macroscopic disease when they received DCRT.

**Table 2:** Patients’ characteristics and tumor information of the five dogs in the DCRT group.

**No.**	**Age (y/o)**	Breed	Sex	Weight (kg)	Tumor diagnosis	Tumor location	Clinical stage	Mass size (cm)^1^	Lymph node metastasis	MC	Bone invasion	Treatments before enrollment	Surgical procedure after enrollment
1	13	Maltese	Fs	2.64	AMM	Lt. maxilla	II	3.4	Yes/path	2/HPF	Yes	Yes, chemo^2^	Biopsy
2	14	Shiba	Mi	13	MM	Lt. mandible	III	3.0	Presumed yes/imaging	6/10 HPF	No	No	Biopsy
3	8	Welsh Corgi	Mc	16.5	MM	Rt. mandible	III	2.1	Yes/cytology	1/HPF	No	No	Biopsy
4	11	Dachshund	Mi	5.8	MM	Lt. maxilla	III	2.5	Yes/path	8/10 HPF	No	No	Biopsy
5	12	Miniature poodle	Mi	4.3	MM	Lt. maxilla	II	4.1	No/path	NR	Yes	No	Biopsy

^1^Maximum diameter was recorded. ^2^Metronomic chemotherapy was prescribed for 2 weeks before DCRT treatment. Fs, female spayed; Mc, Male castrated; Mi, Male intact; AMM, amelanotic malignant melanoma; MM, malignant melanoma; HPF, high power field; path, histopathology; MC, mitotic count; NR, not reported.

Group 2/DC group: 11 dogs were prospectively included. Detailed patient information is summarized in Table 3. The median age of the 11 dogs was 13 years old (IQR, 10–14). The median body weight was 6.8 kg (IQR, 5.7–19). The median oral tumor size was 2.3 cm (IQR, 1.8–3.7). Five dogs (45%) had maxillary tumors, three (27%) had mandibular tumors, and three (27%) had lingual tumors. Three dogs (27%) were stage I/II, and eight (73%) were stage III/IV. Five dogs (45%) had metastatic tumor cells in the RLNs. The median mitotic count was 12/10 high-power fields (HPFs) (IQR, 8–34), ranging from 4 to 102/10 HPFs. Before enrollment, seven dogs (64%) were treatment-naïve; two (18%) had marginal excision but the tumor recurred within 4 weeks; one (9%) received marginal excision and metronomic chemotherapy for 8 weeks then the tumor recurred; one (9%) received metronomic chemotherapy for 6 weeks without clinical benefit. When receiving the DC vaccine, five dogs (45%) had macroscopic disease, either in the oral cavity or the lung parenchyma.

**Table 3:** Patients’ characteristics and tumor information of the 11 dogs in the DC vaccine group.

**No.**	**Age (y/o)**	**Breed**	Sex	Weight (kg)	Tumor diagnosis	Tumor location	Clinical stage	Mass size (cm)^1^	Lymph node metastasis	MC/10HPF	Bone invasion	Treatments before enrollment	Surgical procedure after enrollment
1	14	Shi Tzu	Mc	5.5	MM	Lt. mandible	IV	2.7	Yes/path	37	Yes	No	Oral mass debulking
2	13	Miniature poodle	Fs	5.8	MM	Lt. caudal maxilla	III	4	Yes/path	8	Yes	No	Oral mass partial debulking^2^
3	14	Schnauzer	Fs	6.5	MM	Rt. maxilla	II	2.3	Not reported	12	Yes	No	Oral mass debulking
4	13	Mixed breed	Mc	20	MM	Lingual	III	3.4	Yes/path	15	No	No	Oral mass debulking
5	10	Mixed breed	Mc	21	MM	Lingual	IV	2	No/path	Not reported	No	Marginal excision	Oral mass debulking
6	11	Miniature poodle	Mc	2.8	MM	Lt. maxilla	IV	0.7	No/path	8	Yes	Marginal excision	Partial maxillectomy
7	10	Mixed breed	Mc	20	MM	Lingual and lip	I	1.6	No/path	34	No	No	Glossectomy, lip mass debulking
8	15	Mixed breed	Fs	18.5	MM	Rostral maxilla	III	2.1	Yes/path	Hard to evaluate^3^	No	No	Oral mass debulking
9	14	Mixed breed	Fs	15	AMM	Lt. mandible	III	4	No/path	4	No	Metronomic chemotherapy	Oral mass debulking
10	10	Dachshund	Mi	6.8	MM	Rostral maxilla	III	5	Yes/path	102	Yes	Metronomic chemotherapy	Biopsy
11	8	Miniature poodle	Fi	2.5	MM	Rostral mandible	I	1.3	No/cytology	7	Yes	No	Rostral mandibulectomy

^1^Maximum diameter of the oral tumor; ^2^Oral mass partial debulking: patient No. 2 had the tumor invade the orbital and retrobulbar area, which was not able to be surgically removed. ^3^According to the histopathological report, the mitotic count was difficult to observe due to the obscurations by large numbers of cytoplasmic granules. Fs, female spayed; Fi, female intact; Mc, male castrated; Mi, male intact; MM, malignant melanoma; AMM, amelanotic malignant melanoma; path, histopathology; MC, mitotic count; HPF, high power field.

Group 3: eight dogs were retrospectively collected. Detailed patient information is summarized in Table 4. The median age of dogs in this group was 13.5 years old (IQR, 11.8–14). The median body weight was 9.0 kg (IQR, 7.6–10.4). The median oral tumor size was 2.8 cm (IQR, 3.4–3.1). Four dogs (50%) had mandibular tumors, three (37.5%) had maxillary tumors and one (12.5%) had a tonsil melanoma. Two dogs (25%) were in clinical stage I/II and six (75%) were in stage III/IV. Four dogs (50%) had metastatic RLNs. Two dogs had tumor debulking surgery before receiving carboplatin while the other six dogs were treated in macroscopic disease status. Neither of the dogs in this group received other systemic treatments before carboplatin. None of them received wide-margin surgery or radiotherapy during their disease course.

**Table 4:** Patients’ characteristics and tumor information of the eight dogs in the carboplatin control group.

**No.**	**Age (y/o)**	**Breed**	Sex	Weight (kg)	Tumor diagnosis	Tumor location	Clinical stage	Mass size (cm)^1^	Lymph node metastasis	MC	Bone invasion	Treatments before enrollment	Surgical procedure
1	14	Schnauzer	Mi	6.9	MM	Lt. mandible	III	2.5	Yes/path	30/10 HPF	No	Marginal excision	Oral mass debulking
2	14	Schnauzer	Mc	9.1	MM	Rt. Mandible	II	3.6	No/cytology	8–10/10 HPF	Yes	Marginal excision	No
3	14	Dachushund	Fs	8.8	MM	Rt. Maxilla	I	2.0	No/cytology	0-2/HPF	Not reported	Marginal excision	No
4	11	Schnauzer	Mc	6.3	MM	Maxilla/soft palate	III	4.5	Yes/cytology	0-2/HPF	Not reported	No	No
5	15	Beagle	Mi	20	MM	Rt. Maxilla	IV	1.5	Not reported	Not reported	Not reported	Marginal excision	No
6	13	Scottish terrier	Mi	13	MM	Tonsil	IV	2.6	Yes/path	5-8/HPF	No	No	No
7	10	Schnauzer	Fs	7.8	MM	Rt. Mandible	IV	3.0	No/cytology	5-7/HPF	Yes	Marginal excision	No
8	12	Shiba	Mi	9.5	MM	Rt. Mandible	III	3.0	Yes/imaging	Rare	No	No	Oral mass debulking

^1^Maximum diameter of the oral tumor; Fs, female spayed; Mc, male castrated; Mi, male intact; MM, malignant melanoma; path, histopathology; MC, mitotic count; HPF, high power field.

Patient characteristics between the three groups were not significantly different.

### 3.2 Treatment response and adverse events

Group 1/DCRT group: results of the five dogs are summarized in Table 5. Four dogs received DCRT following the planned schedule and protocol. One patient (No. 4) received RT on schedule but started the DC vaccination after the third radiation because, under histopathological exam, only rare cells contained pigments, and additional immunohistochemistry stains were required to confirm the melanoma diagnosis. All dogs responded to radiation therapy, two had CR and three had PR as the best response. Regarding RT toxicity, only mild and self-limited acute side effects were observed, including grade 1 alopecia and grade 2 mucositis. For the DC vaccination, three dogs did not report any side effects, one dog had a tiny, self-recovered injection site subcutaneous nodule that was too small and deep to perform a fine-needle aspiration (FNA), and the other had grade 1 hyporexia. No aggressive medical intervention was needed in this group.

**Table 5:** Treatment protocol, adverse events and outcomes of the five dogs in the DCRT group.

**No.**	**RLN removal**	RT (oral mass)	RT (RLN)	DC doses	Best response	Adverse events (RT/DC)	PFI (days)	TLP (days)	TDM (days)	OST (days)	Other treatments after PD	Outcome
1	Yes	8.5 Gy*5	No	Regular, 4 doses	CR	Grade 2 mucositis, grade 1 skin/NR	79	79	No	113	No	Spontaneous death; local disease
2	No	8 Gy*5	7.5 Gy*5	Regular, 4 doses	PR	Grade 2 mucositis/NR	62	102	62; pulmonary	62	Metronomic chemotherapy	Spontaneous death; pulmonary metastasis
3	No	8 Gy*5	7 Gy*5	Regular, 4 doses	CR	Grade 1 skin/Injection site nodule	630^2^	No	Unsure	630	Progression was not confirmed	Spontaneous death; liver failure, neurological signs
4	Yes	8 Gy*5	8 Gy*5	started after 3^rd^ RT, 4 doses^1^	PR	Grade 1 skin/Grade 1 hyporexia	101^2^	Unsure	Unsure	101	Progression was not confirmed	Sudden death; undetermined cause
5	Yes	8 Gy*5	7 Gy*5	Regular, 4 doses	PR	Grade 1 skin/NR	214	214	No	214	Adoptive NK cell therapy	Spontaneous death; local disease
Median PFI (days)						214 (95% CI, NA)						
Median OST (days)						Not reached due to insufficient event numbers						

1: the patient started DC vaccination after the third radiation because additional immunohistochemistry stains were required to confirm the final melanoma diagnosis. 2: patient No. 3 died of liver failure and neurological signs without evidence of local tumor recurrence or pulmonary metastasis, but liver or brain metastasis could not be ruled out; patient No. 4 died of undetermined cause, the patient developed occasional vomiting and coughing around two weeks before death (four weeks after treatment finished), a tumor-related death could not be excluded but was not confirmed. The data were recorded as durations from treatment to death. RLN, regional lymph node; NR, not reported; PFI, progression-free interval; TLP, time to local progression since treatment started; TDM, time to distant metastasis since treatment started; OST, overall survival time; NK cell, natural killer cell.

Group 2/DC group: results are summarized in Table 6. All 11 dogs had sufficient tumor cell counts and were scheduled to receive four vaccination doses. Nine dogs finished the full protocol, and two of them extended the treatment to a total of five doses, as the owners required. Two dogs (patients No. 4 and 10) did not finish the treatment because of tumor progression, after receiving two and three doses of DC vaccine, respectively. No dog responded to the treatment, while eight dogs achieved stable disease (three were stable with macroscopic lesions, five were progression-free with microscopic tumor cells) during vaccination treatment, resulting in a clinical benefit rate of 73%. Two of the three stage IV dogs could maintain stable disease during treatment and survived for over a year. Nine dogs did not report any adverse events during the whole treatment process. One dog had a 1-cm injection site nodule and FNA revealed predominantly neutrophils and macrophages. This patient had a self-recovery without medical intervention. The other dog reported grade 2 gastrointestinal toxicities, including hyporexia, vomiting and diarrhea, which could be managed by supportive treatments.

**Table 6:** Treatment protocol, adverse events and outcomes of the 11 dogs enrolled in the DC vaccine group.

**No.**	**RLN removal**	**DC doses**	Residue tumor before DC vaccination	Best response	Adverse events	PFI (days)	TLP (days)	TDM (days)	OST (days)	Treatments after PD	Outcome
1	Yes	4	Macroscopic/lung	SD	NR	161	No	161; pulmonary PD	161	Chemotherapy; ACT	Euthanasia; pulmonary metastasis
2	Yes	5	Macroscopic /retrobulbar	SD	Injection site nodule	70	70	141; pulmonary	70	ACT; surgery	Euthanasia; local disease
3	No	5	Microscopic	SD/PF	NR	117^2^	177	204; pulmonary	117	ACT; TKI; chemotherapy	Spontaneous death; Local lymphadenomegaly; SIRS, AKI
4	Yes	2	Microscopic	PD	NR	14	14	21; pulmonary	23	No	Euthanasia; diffuse metastasis
5	Yes	4	Macroscopic/lung	SD	grade 2 GI signs^1^	100^2^	333	366; pulmonary PD	100	ACT	Spontaneous death; pulmonary metastasis
6	Yes	4	Macroscopic /lung	PD	NR	31	31	31; pulmonary PD	51	No	Euthanasia; diffuse metastasis
7	Yes	4	Microscopic	SD/PF	NR	418	No	418; pulmonary	525	No	Euthanasia; pulmonary metastasis; UB mass, hematuria; liver and splenic mass; anemia;
8	Yes	4	Microscopic	SD/PF	NR	686^3^	No	No	686	No	Spontaneous death; anorexia; Rt. forelimb mass
9	Yes	4	Microscopic	SD/PF	NR	60	60	No	60	TKI	Euthanasia; local tumor, AKI, cachexia;
10	Yes	3	Macroscopic /oral	PD	NR	27	27	No	44	No	Euthanasia; local disease;
11	No	4	Microscopic	SD/PF	NR	237	No	237; subcutaneous 316; pulmonary	237	Chemotherapy; TKI	Spontaneous death; diffuse distant metastasis
**Median PFI (days)**					100 (95% CI, 27–237)						
**Median OST (days)**					525 (95% CI, 44-NA)						

^1^Gastrointestinal (GI) signs, including hyporexia, vomiting, and diarrhea. ^2^Patients did not have disease progression at this time. Owners insisted on continuing with other treatments, so the PFIs were recorded as the duration from DC vaccination to initiation of other treatments. ^3^Patient No. 8 had a rapidly enlarged Rt. forelimb mass 19 months after DC finished and died within 2 months, without evidence of tumor progression. Melanoma-related death could not be ruled out but was not confirmed. The data was recorded as the duration from treatment to death. RLN, regional lymph node; PF, progression-free; NR, not reported; PFI, progression-free interval; TLP, time to local progression since treatment started; TDM, time to distant metastasis since treatment started; OST, overall survival time; ACT: adoptive natural killer cell therapy; TKI, tyrosine kinase inhibitor; SIRS, systemic inflammatory response syndrome; AKI, acute kidney injury; UB, urinary bladder.

Group 3: results are presented in Table 7. The median carboplatin initiation dosage of all eight patients was 250 mg/m^2^ (IQR, 250–262.5) and the median dose of injections was 2 (IQR, 1–3). One dog had a partial response to carboplatin, and four maintained stable disease, leading to a 12.5% response rate and 62.5% clinical benefit rate. Side effects were reported in five dogs which were mainly grade 1–2 and self-limited gastrointestinal discomfort.

**Table 7:** Treatment protocol, adverse events and outcome of the eight dogs in the carboplatin control group.

**No.**	RLN removal	Residue tumor before chemotherapy	Chemotherapy	Best response	Adverse events	PFI (days)	TLP (days)	TDM (days)	OST (days)	Treatments after PD	Outcome
1	Yes	Microscopic	Carboplatin; 250 mg/m^2^; 3 cycles	SD	Grade 1–2 GI^1^	169	176	169	211	No	Spontaneous death; pulmonary metastasis
2	No	Macroscopic	Carboplatin; 250 mg/m^2^; 2 cycles	SD	Grade 1–2 GI^1^	44	44	No	197	No	Euthanasia; local disease
3	No	Microscopic	Carboplatin; 250 mg/m^2^; 3 cycles	PF	Grade 1–2 GI^1^; grade 2 ALT elevation	PF	PF	PF	Alive	No	Follow up: > 960 days without progression
4	No	Macroscopic	Carboplatin; 250 mg/m^2^; 1 cycle	PR	NR	42	42	No	42	No	Spontaneous death; local disease
5	No	Macroscopic	Carboplatin; 300 mg/m^2^; 1 cycle	NA	NA	21	NA	NA	21	NA	Unsure; died 21 days after chemotherapy
6	Yes	Macroscopic	Carboplatin; 300 mg/m^2^; 3 cycles	SD	NR	31	31	98	100	No	Spontaneous death; pulmonary metastasis
7	No	Macroscopic	Carboplatin; 300 mg/m^2^; 1 cycle	PD	Grade 1 GI^1^	14	14	14	81	No	Spontaneous death; pulmonary metastasis, anorexia
8	No	Macroscopic	Carboplatin; 250 and 300 mg/m^2^	PD	Grade 1 creatinine elevation	28	28	No	28	Immunotherapy	Spontaneous death; local disease
Median PFI (days)				42 (95% CI, NA-170)							
Median OST (days)				148.5 (95% CI, NA)							

^1^Gastrointestinal (GI) signs, including hyporexia, vomiting, and diarrhea. RLN, regional lymph node; PF, progression-free; NA, not assessed; NR, not reported; PFI, progression-free interval; TLP, time to local progression since treatment started; TDM, time to distant metastasis since treatment started; OST, overall survival time.

### 3.3 Outcome

All dogs in Groups 1 and 2 had died by the time of this manuscript preparation.

In the DCRT group, three dogs died because of tumor-related reasons (two died of local disease, one was due to distant metastasis), and two of them received other kinds of treatments (other immunotherapies, metronomic chemotherapy) after tumor progression. One dog (No. 3) survived 22 months and died of liver failure with neurological signs. No local recurrence or pulmonary metastasis was observed before death; however, whether the liver or brain had melanoma metastasis, or the dog developed primary liver diseases, was not confirmed. Treatment-related reasons were less likely due to the long duration from treatment to death, and no adverse events were reported during treatment. The other dog (patient No. 4) had a sudden death 28 days after treatment finished, without any evidence of disease progression at the last visit. However, tumor-related reasons could not be excluded because the dog had occasional vomiting and coughing 2 weeks before death. Although the activity, appetite and respiratory rate were normal, melanoma metastasis or tumor emboli were possible causes of death. Treatment-related reasons were less likely because no abnormal radiation-induced or immuno-dysregulation signs were noted. Other diseases, including cardiopulmonary or gastrointestinal problems, were not excluded either, but no more clinical signs were reported. No necropsy was performed.

In the DC group, all 11 dogs had died. Four (36%) deaths were because of local tumor progression, six dogs (55%) died of distant melanoma metastasis; one dog (patient No. 8) developed a rapidly enlarged Rt. forelimb mass around 19 months after DC finished, and became anorexia and died within 2 months. The owner declined diagnostic exams of the Rt. forelimb mass, therefore, whether the mass was a melanoma metastasis or a second malignancy was undetermined, or if there were other comorbidities leading to the patient’s death. Six dogs received other treatments after disease progression (other immunotherapies, chemotherapy, or TKIs). After the DC treatments were finished, two dogs received other immunotherapies without disease progression due to the owners’ insistence. The PFIs in these two dogs were recorded as the duration from the day DC vaccination started to the time of other treatment initiation. Among the three stage IV dogs, patient No. 1’s pulmonary lesion was solitary and 0.3 cm in diameter, and could only be detected on thoracic CT. The nodule progressed 5 months later after DC vaccination. The patient then received other treatments (immunotherapy, chemotherapy) and survived another 8 months, and died of extensive pulmonary metastasis. Patient No. 5, with stage 4 lingual melanoma, also had a stable solitary pulmonary lesion, and the owner wanted to continue with another immune cell therapy using autologous natural killer cells after finishing DC vaccination. The pulmonary lesion achieved PR after cell therapy. However, the patient developed local recurrence 8 months later, and the pulmonary metastasis deteriorated 9 months later. Patient No. 6 had two tiny pulmonary nodules measured about 0.2 cm on thoracic CT before treatment, and presented with an extensive miliary to nodular pulmonary metastatic pattern on chest X-ray before the third DC treatment. The patient also developed suspected brain metastasis and was euthanized 2 months later. Patient No. 11 developed diffuse subcutaneous masses around 8 months after treatment started, and the masses were confirmed to be melanoma by histopathology. The patient then received chemotherapy (carboplatin, doxorubicin) and targeted therapy and survived 5 months more.

In Group 3, six dogs died because of tumor progression (three died of local disease, three died of pulmonary metastasis); one dog (patient No. 5) died 21 days after treatment, the cause was undetermined and either rapid tumor progression or treatment-related fatal adverse events were possible reasons. Seven dogs had carboplatin as their sole systemic treatment, while one dog received immunotherapy after carboplatin failure but no obvious response was observed. Patient No. 3 was still alive without tumor recurrence at the time of data collection (follow-up time, 960 days).

**Figure 2:**
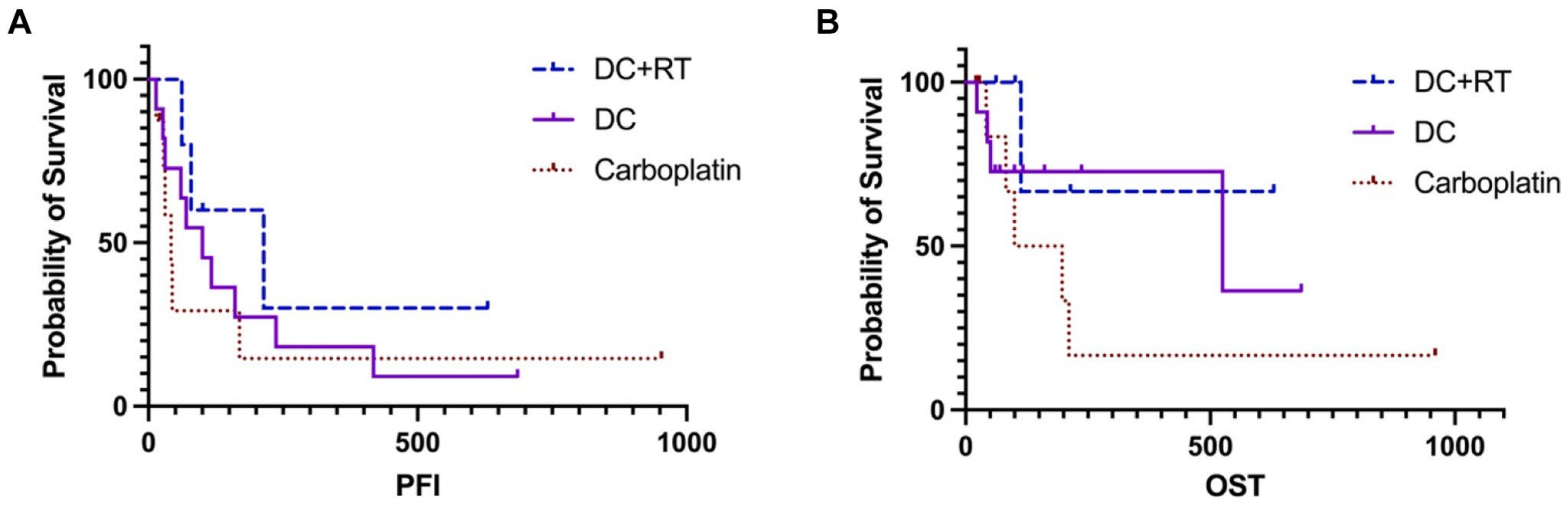
The Kaplan–Meier curves of PFI **(A)** and OST **(B)** survivals in the DCRT and DC groups. Neither revealed significant differences. The censored data were presented as vertical tick marks.

For survival analysis, in the DCRT group, patients No. 3 and 4 were censored from PFI analysis because tumor progression was not confirmed, leaving the PFI of 79, 62 and 214 days in the other three dogs; the median PFI was 214 days (95% CI, NA). Regarding the overall survival, patients No. 3 and 4 were censored from OST analysis because of undefined causes of death, and patients No. 2 and 5 were censored because they received other treatments after failing DCRT; survival times of these dogs were recorded (Table 5) but median OST could not be reached due to insufficient uncensored data. Overall, the 1-year survival rate was 20% in the DCRT group. In the DC group, patient No. 8 was censored from PFI analysis, resulting in a median PFI of 100 days (95% CI, 27–237). Seven dogs were censored from OST analysis because of unidentified tumor-related death (patient No. 8) or receiving other treatments after tumor progression (patients No. 1, 2, 3, 5, 9, 11). The median OST was 525 days (95% CI, 44-NA), and the 1-year survival rate was 54.5%. In the carboplatin group, two dogs were censored from PFI analysis (patient No. 3 was alive, and patient No. 5 had an undetermined cause of death), resulting in a median PFI of 42 days (95% CI, NA-170). Three dogs were censored from OST analysis (patients No. 3, 5, and 8), leading to a median OST of 148.5 days (95% CI, NA); the 1-year survival rate was 12.5%. The PFI and OST are presented by the Kaplan–Meier curves in Figure 2. The survival curves were not significantly different by the Log-rank test.

### 3.4 Cytokine analysis

All patients in the DCRT group and six in the DC group had sufficient plasma collection at different time points for cytokine analysis. Some concentrations of the evaluated cytokines were too low to be detected. After discussing with the product specialist, we decided to use both concentration and median fluorescent intensity (MFI) for those cytokines with detectable concentration values, while for those the concentration values were non-detected, MFI was used for analysis.

No clear association between cytokine changes and clinical evolution was found. In the DCRT group, no cytokine changed significantly before or after treatment. While in the DC group, although no cytokine revealed significant change, the MCP-1 increased after treatment (*p* = 0.06), and the VEGF-A decreased (p = 0.06).

Between the two groups, the TGF-β and IFN-γ MFIs were significantly higher in the DCRT group. The TGF-β MFI was higher in the DCRT group before DC vaccination (*p* = 0.004), and remained higher during (*p* = 0.016) and post-vaccination (*p* = 0.036), compared to the DC group. While the IFN-γ MFI was significantly higher during (p = 0.016) and after vaccination (*p* = 0.018) in the DCRT group.

## 4 Discussion

The current manuscript reported a pilot study investigating the safety and efficacy of dendritic cell/tumor cell fusion vaccine immunotherapy, alone or in combination with radiotherapy, in treating canine oral malignant melanoma. Safety evaluation was the primary study aim, which indicated that both DC and DCRT were well-tolerated, with only mild gastrointestinal and injection site adverse events recorded. Overall, dogs that received the DC vaccine achieved a median PFI of 100 days (95% CI, 27–237), while dogs in the DCRT group had a median PFI of 214 days (95% CI, NA). Dogs in the DC group seemed to live longer and had a higher 1-year survival rate (54.5 vs. 20%). However, compared to the retrospectively collected control group composed of dogs receiving carboplatin without wide-margin surgery or RT, treating with DC or DCRT did not generate significantly superior survivals. But at least the general safety of DC vaccination preliminarily supported further exploration of this treatment in a more rigorously constructed clinical trial to assess its efficacy. It is to be noted that chemotherapy is not a standard of care in dogs with malignant melanoma. The authors chose this group because of the current treatment dilemma of lacking RT and other immunotherapies in our area, resulting in the necessity of investigations on treatments other than surgery. In addition, the authors did not enroll dogs receiving only surgery as a control group because those dogs in our department were predominantly in clinical stage I-II, which might carry an inherently better prognosis than dogs in the DCRT or DC group. As for dogs receiving only RT, the medical records were not as complete as we required.

The PFI is the appropriate statistic to evaluate treatment efficacy. Median PFI was only 100 days in the DC group. However, two patients switched to another immunotherapy after completing the DC vaccination without disease progression, potentially underestimating the actual PFI. Notably, two of the three stage IV dogs (patients No. 1 and 5) survived for over a year and could maintain stable disease during DC vaccination. The pulmonary metastases were solitary and small in these two dogs, which might facilitate the immunotherapy to work, as the tumor burden was relatively not heavy. Moreover, in patient No. 5, the subsequent adoptive natural killer cell therapy after DC vaccination resulted in partial remission of the pulmonary metastasis, but we did not design a study to explore the exact mechanism and efficacy of combining the two immunotherapies. Lacking wide-margin surgery and RT resulted in inadequate local control in most dogs in the DC group. Only patient No. 8 had the least tumor burden by removing the primary oral tumor and two metastatic lymph nodes and survived 23 months without additional treatments. Patient No. 2 had mild orbital invasions that could not be removed by surgery and had a modest survival time; patient No. 10 carried an invasive primary oral mass and experienced a worse outcome; patient No. 4 had the most aggressive tumor that progressed dramatically despite treatment intervention. A future clinical study of using DC vaccination as an adjuvant therapy could be considered.

However, the clinical outcome of the DCRT group did not meet our expectations. Firstly, although the case number was low, the percentage of dogs with RLN metastasis was numerically higher in the DCRT group, indicating a possible poorer prognosis. Besides, in theory, radiation can induce tumor cell death, leading to tumor-antigen release and subsequent antigen-presenting process, alteration in tumor surface markers (e.g., MHC-I, FAS ligand, immune checkpoint molecules) expression, production of cytokines and chemokines, and recruitment of CD8 T cells and tumor-infiltrating lymphocytes, all of which exerts the immune-stimulation facility. Meanwhile, radiation also has an immunosuppressive impact by increasing Treg infiltration, TGF-β and IL-10 excretion, and myeloid-derived suppressor cells (MDSCs) recruitment (23, 24). Therefore, our initial hypothesis for this inferior DCRT outcome is the potential RT-induced immunosuppressive environment, as we found that compared to the DC group, the MFI of TGF-β in the DCRT group was significantly higher throughout the treatment course. The MFI of IFN-γ was also significantly higher in the DCRT group after treatment. Radiation-induced IFN-γ production can activate APCs and T cells and promote a tumor-killing process; on the other hand, IFN-γ can also induce PD-L1 expression and stimulate tumor prosurvival mechanisms (23). We should not use this data to draw a conclusion, as changes in immune function and the correlated immunotherapeutic clinical outcomes should not be interpreted using a single parameter or a simple combination. However, further exploration of those cytokines’ functions in a larger group is worthwhile, as well as using a comprehensive complex of immune response patterns consisting of lymphocyte recirculation and subpopulation activations, APCs’ function changes, along with the cytokines profiles, to elucidate the correlations between immune pattern changes and clinical outcomes.

The DCRT treatment sequence should be investigated. In our protocol, due to the DC vaccine manufacturing process and some force majeure related to RT, we overlapped RT and DC vaccination, as the vaccination started after the second radiation fraction. It is not clearly understood which treatment sequence is optimal or at what exact time point immunotherapy should be inducted. A study by Deguchi et al. (31) was the only one that compared different treatment schedules of ICI and RT in dogs (31), and they found that the best outcome was achieved in the previous RT group (dogs treated with RT within 8 weeks prior to the first ICI dose) rather than the concurrent RT group (ICI was given within 1 week of the first RT). Although most of the current studies focus on a combination of ICIs and RT, and the different mechanisms of ICIs and the DC vaccine require different evaluations of treatment sequence, it is still possible that our DCRT protocol was suboptimal. Because we gave the DC vaccination after the second RT, some dogs exhibiting partial responses to the first RT may have less tumor antigen release and an inferior antigen-presenting process in the second RT. Further studies could be designed to give the DC vaccine on the same day as the first RT, to see if there is a better outcome.

Besides, at the time we designed our DCRT protocol, there was not much study discussing the regional lymph node impact, and the inclusion of RLNs in the radiation field was determined by each clinician. Recently, studies in mouse models with head and neck tumors have shown that elective lymph node irradiation ablated the combinatorial efficacy of SBRT and immunotherapy and reduced antigen-experienced T cell expansion (39). Similar findings were also observed in dogs with nasal tumors, that nodal irradiation reduced the CD4 and CD8 T cell counts in the nasal cavity, reduced gene expressions in antigen presentation and T cell activation, and increased immunosuppressive gene expressions (39). Another team reported similar findings that lymphablation was deleterious to ICI response and overall survival in mouse models, and the DCs in draining lymphatics were necessary for the ICI response (40). In light of these results, we found that four out of five dogs in our DCRT group had regional lymph nodes included in their RT fields. Although we did not irradiate all the draining lymph nodes but only included one or two of the RLNs, it may still impact the immunotherapeutic effect adversely but failed to be significantly presented due to the small case number. However, canine OMM has a higher nodal metastatic rate than sinonasal tumors. For those RLNs that have abundant metastases and are structurally effaced by tumor cells, whether to spare these nodes during RT may need further evaluation, as the nodal microenvironment and immune cell composition might differ. Alternatively, the metastatic RLNs could be preserved initially to generate immune responses during RT and immunotherapy, and be removed later. According to Darragh et al. (39) study, sentinel lymphadenectomy after treatment completion led to a decreased local metastatic rate and had no impact on systemic immunity. More clinical trials are warranted regarding these questions.

The DC generation and fusion vaccine preparation methodology followed established protocols (18, 19), with clear functional and immunophenotypic evaluation. Based on the previous studies, the morphology of the PBMC-generated DCs was similar to typical DCs; the phenotypic expressions of CD80, CD83, CD86, CD1a, CD11c, CD40, and MHC II were observed in mature DCs, as were productions of IL-1b, IL-10, IL-12p40, IL-13, and TNF-α. Similar to previous studies, the DC generation rate in the current study was calculated as 3–4 × 10^7^ mature DCs per 100 mL of peripheral blood and was uniform among different generating processes. Therefore, we did not repeat the function and expression analyses, and only confirmed the DCs’ morphology microscopically. Our previous experiments also showed that treating dogs with OMM with the BMDC/tumor cell fusion vaccine and surgery significantly prolonged survival, decreased circulating Tregs and increased melanoma-specific cytotoxicity were also observed (34). Allogeneic DCs and autologous tumor cell fusion products can stimulate immune reactions directly or via cross-presentation through the expressions of DC-derived MHC-I, DC-derived MHC-II loading with tumor antigen, and tumor-derived MHC-I loaded with tumor antigen (15). Cross-presentation of allogeneic DCs to the host could allow antigens to be presented by the host’s antigen-presenting cells, priming the immune response (21). Although different epitopes from allogeneic DCs can stimulate allorecognition, it has been suggested that the MHC molecules should be partially shared between donor and host to generate antigen-specific T-cell responses (15, 41). In this manuscript, we did not analyze the MHC allele similarity between our patients and donor dogs. Thus, mismatches may exist, potentially leading to a lack of tumor antigen-specific immunostimulation and weakened DC vaccination efficacy. Nevertheless, we still chose to use allogenic sources because autologous DCs from cancer-bearing patients may be defective; besides, dogs in our study were predominantly small to medium-sized populations, and it was harmful to collect 50 or 100 mL of peripheral blood or perform a bone-marrow aspiration to generate sufficient mononuclear cells for DC vaccine preparation. Moreover, by recognizing and presenting the MHCs of allogeneic DCs, as well as the MHCs of tumor-bearing dogs in the fusion cells, it was theoretically reasonable that T-lymphocytes could still be stimulated by this method, generating the expected immune response.

Except for the questions mentioned above, several limitations exist in this manuscript. First, the sample size was small. One of the obstacles in patient recruitment was an inherent feature of using autologous tumor cells. Because we harvested primary tumors from canine oral cavities, it was unsurprising that bacterial pollution existed during the cell culture process, rendering an exclusion from the treatment.

Furthermore, the treatment outcome could not be rigorously compared because this was not a double-blind random prospective clinical trial, and some deviations existed in the patient recruitment and treatment process. For example, the clinical stage inclusion criteria were mildly different between DC and DCRT groups, because RT was not strongly suggested if the patient was in stage IV, but the early pulmonary metastasis, which was detected by CT, was allowed in the DC group. Besides, at the time of DCRT patient enrollment, there was no consensus among our clinicians regarding the RLN removal or RLN inclusion in the RT field, resulting in inconsistent decisions in the studied population. Thirdly, as a pilot study, we allowed patients who were previously treated to enroll, and patients who failed DC or DCRT to switch to other therapies, which was also a confounder on efficacy evaluation and resulted in a largely censoring process on OST analysis. Specifically speaking, before starting DC or DCRT, three dogs had metronomic chemotherapy with a duration from 2 weeks to 2 months. As metronomic chemotherapy can generate antineoplastic effects through inhibitions on angiogenesis and Tregs accumulation, and induction of tumor dormancy (42), whether these three dogs had immune modulations which then affected the treatment efficacy, was undetermined. In addition, although the DC protocols in the two groups were generally uniform as designed, one dog in the DCRT group started DC vaccination before the third RT, and two dogs in the DC group extended to five doses as owners required. Whether those deviations affected outcomes was also unknown. Lastly, examinations of the abdominal cavity and urinalysis were not routinely required, leading to a possible underdiagnosis; and some histological characteristics, such as the mitotic figures, were not consistently calculated by 10 HPFs in the DCRT group. However, a prognostic cut-off value was ≥4/10 HPFs (43), and most of our patients had mitotic counts exceeding this value, or in those with unmentioned or calculated mitoses per HPF, metastatic disease was diagnosed, indicating a poorer prognosis in general.

## 5 Conclusion

The allogeneic DC and autologous tumor cell fusion vaccine immunotherapy alone and in combination with local radiotherapy reported in this manuscript were well-tolerated in dogs with oral melanoma. Receiving the DC vaccine alone or the combinatorial DCRT did not demonstrate a survival difference but the cytokine analysis revealed a higher TGF-β signal in the DCRT group, indicating a potential immunosuppressive status. Given the general safety and the limitations of the study design, the current manuscript supports the further need for a more well-constructed clinical trial on an expanded scale, to investigate the actual efficacy of DC and DCRT in dogs. Studies on treatment sequence and protocol modification in the DCRT group, as well as analysis of the peripheral blood immune cells and cytokines, are also warranted.

## Data Availability

The original contributions presented in the study are included in the article/supplementary material, further inquiries can be directed to the corresponding author.

## Footnotes

^1^ www.graphpad.com
